# A Role for Progesterone-Regulated sFRP4 Expression in Uterine Leiomyomas

**DOI:** 10.1210/jc.2016-4014

**Published:** 2017-06-14

**Authors:** Meaghan A. Delaney, Ying-Wooi Wan, Gyoung-Eun Kim, Chad J. Creighton, Margaret G. Taylor, Ramya Masand, Andrew Park, Cecilia Valdes, William Gibbons, Zhandong Liu, Matthew L. Anderson

**Affiliations:** 1 Department of Obstetrics & Gynecology, Baylor College of Medicine, Houston, Texas 77030; 2 Department of Molecular and Human Genetics, Baylor College of Medicine, Houston, Texas 77030; 3 Dan L. Duncan Comprehensive Cancer Center, Baylor College of Medicine, Houston, Texas 77030; 4 Department of Pathology & Immunology, Baylor College of Medicine, Houston, Texas 77030; 5 Department of Pediatrics, Baylor College of Medicine, Houston, Texas 77030

## Abstract

**Context:**

Despite progesterone’s key role in uterine smooth muscle tumorigenesis, the mechanisms by which it promotes the growth of uterine leiomyomas remain poorly understood.

**Objective:**

The aim of this study was to identify gene products mediating the effects of progesterone in uterine leiomyomas.

**Design:**

Gene expression profiling was used to identify putative progesterone-regulated genes differentially expressed in uterine leiomyomas, which were then studied *in vitro*.

**Methods:**

Gene expression was comprehensively profiled with the Illumina WG BeadChip (version 2.6) and analyzed with a bioinformatic algorithm that integrates known protein–protein interactions. Genomic binding sites for progesterone receptor (PR) were interrogated by chromatin immunoprecipitation—quantitative polymerase chain reaction (ChIP-qPCR). Small interfering RNA was used to study gene function in primary cell lines.

**Results:**

Our analyses identified secreted Frizzled-related protein 4 (sFRP4) as a key gene product functionally linked to PR activation whose expression was 2.6 times higher in leiomyomas than myometrium (n = 26, *P* < 0.01) and 2.5 times higher during the proliferative phase of the menstrual cycle (n = 26, *P* < 0.01). Direct binding between PR and sFRP4 promoter was observed by ChIP-qPCR. Robust overexpression of sFRP4 was also observed in primary cultures derived from leiomyoma. Progesterone preferentially inhibited sFRP4 expression and secretion in leiomyoma cultures in a dose-dependent manner sensitized by estradiol. Knockdown of sFRP4 inhibited proliferation and apoptosis in primary cultures of both myometrium and leiomyoma.

**Conclusions:**

Overexpression of sFRP4 is a robust, progesterone-regulated feature of leiomyomas that increases smooth muscle proliferation. More work is needed to elucidate how progesterone’s ability to modulate sFRP4 expression contributes to uterine smooth muscle tumorigenesis.

## Teaser

Studying patterns of gene expression that vary by menstrual phase, we found that overexpression of sFRP4 is a progesterone-regulated feature of leiomyomas that regulates smooth muscle proliferation.

Proliferations of uterine smooth muscle, known as leiomyomas, can be found in as many as 80% of premenopausal women (1). Uterine leiomyomas are a common cause of irregular vaginal bleeding, pelvic pain, urinary incontinence, and infertility (2). Nearly one-third of all hysterectomies in the United States, >200,000 surgeries annually, are performed to provide relief from pelvic pain and bleeding caused by these benign tumors (3). At present, noninvasive options for medically managing uterine leiomyomas remain limited. However, the need for new medical therapies is underscored by recent concerns that the routine morcellation of uterine masses during their laparoscopic removal can disseminate an unappreciated malignancy. This concern has rapidly altered patterns of care over the past several years and increased the proportion of surgeries performed to manage leiomyomas by more invasive means. In turn, these changes mean that the morbidity and mortality associated with the surgical management of uterine leiomyomas have increased substantially (4).

Leiomyomas are characterized by robust expression of estrogen receptor (ER) and dysregulated expression of multiple other members of the nuclear receptor superfamily (5). Consistent with the key role of steroid hormones in driving leiomyoma growth, gonadotropin-releasing hormone agonists and other interventions designed to suppress steroid hormone activity can be used clinically to shrink leiomyomas and provide symptomatic relief (6). However, the clinical utility of these agents is often limited by inconsistent clinical responses and poorly tolerated side effects.

For many years, estrogen has been widely accepted to be the primary steroid hormone driving leiomyoma growth. More recently, however, multiple observations have suggested that activation of the progesterone receptor (PR) also plays an important role in uterine smooth muscle tumorigenesis. Leiomyomas express PR at significantly higher levels than adjacent normal myometrium (7). Incubation of primary leiomyoma cultures with progesterone *in vitro* has been shown to stimulate proliferation and inhibit apoptosis (8). A role for PR in these tumors is also supported by observations that the growth of human leiomyoma xenografts *in vivo* is directly stimulated by administration of progestins (9). Clinically, the use of selective PR modulators, such as ulipristal acetate or asinopril, shrinks uterine leiomyomas and can provide sustained symptomatic relief for some women (10, 11). However, the reasons why some uterine leiomyomas respond to a selective PR modulator whereas others do not are not currently known. In part, these responses may reflect altered expression of specific cofactors, such as KLF11, known to directly bind PR and modulate its activity (12).

To clarify these issues, investigators have recently begun to delineate the mechanisms by which progesterone affects leiomyoma growth. These efforts have defined tissue-specific consensus PR binding sites and identified specific genes modulated by application of the synthetic antiprogestin mifepristone in primary cultures derived from uterine leiomyomas (13). Several of these gene products, such as adipophilin, have also been shown to promote the proliferation and migration of leiomyoma cells in culture. PR activation has also been shown to promote leiomyoma growth by increasing the synthesis and deposition of extracellular matrix (14). However, given the numerous PR binding sites in the human genome, it can be difficult to discern which of the many gene products potentially regulated by PR is most important for regulating leiomyoma growth. More recently, investigators have implicated PR activation in regulating myometrial side populations with stemlike properties potentially involved in the initiation and progression of leiomyomas (15). Although side populations from uterine smooth muscle lack ER and PR, their coculture with mature myometrial cells treated with estrogen or progesterone promotes differentiation via paracrine activation of Wnt/*β*-catenin signaling. The specific signals mediating this paracrine process remain unclear.

Given the complex and poorly understood role of progesterone in promoting leiomyoma growth, we undertook the current study with the goal of better understanding the molecular mechanisms by which activation of its receptor contributes uterine smooth muscle tumorigenesis.

## Materials and Methods

### Specimen collection and processing

Permission to work with human tissue specimens was obtained from the Institutional Review Board (H-22119, approved 27 May 2008). Flash-frozen specimens of myometrium and leiomyoma were obtained from Gynecologic Tissue Biorepository at Baylor College of Medicine. All leiomyoma specimens were collected from tumors between 3 and 6 cm in largest transverse diameter at sites ≥1 cm deep to the pseudocapsule. Myometrial specimens were collected ≥1 cm from the nearest leiomyoma. Any specimens collected from women treated with oral contraceptives or medroxyprogesterone in the 4 months before surgery were excluded. Menstrual dating was performed according to established histologic criteria to evaluate each patient’s endometrium by using corresponding hysterectomy specimens (16). All histologic assessments were made by experienced board-certified anatomic pathologists (R.M.). Written informed consent was available for all specimens used.

### Gene expression profiling and analysis

Total RNA was prepared with the miRVana kit (Ambion/Life Technologies, Carlsbad, CA) as previously described (17). Patterns of gene expression were interrogated with the Illumina WG BeadChip (version 2.6). This procedure was performed as a fee for service by the Genome Profiling Core of the Texas Children’s Hospital Cancer Center. Quantile normalized gene expression of gene products was then compared via two-way analysis of variance (ANOVA) with multiple hypothesis adjustment by the Benjamini–Hochberg procedure (18). Unsupervised *K*-mean clustering (*k* = 2) was used to evaluated subsets of specimens for outliers. Significant dysregulated genes (adjusted *P* < 0.05) whose expression varied ≥1.5 times between menstrual phases were used to delineate the functional networks of hormone-responsive genes with GeneMANIA software (www.genemania.org, last accessed 13 May 2014).

### Real-time quantitative polymerase chain reaction

To create complementary DNA, 100 ng of RNA from each specimen was reverse transcribed with the qScript cDNA SuperMix kit (Quanta Biosciences, Gaithersburg, MD). Expression of secreted Frizzled-related protein 4 (sFRP4), PGR, Hes6, sFRP5, ESR1, and ITPKA was evaluated via validated assays to perform real-time quantitative polymerase chain reaction (RT-qPCR) using TaqMan Universal Master Mix II (Applied Biosystems, Foster City, CA). Expression of all other genes was evaluated with SYBR Green Mastermix (Thermo Fisher Scientific, Waltham, MA). Primers used to perform these assays are described in Supplemental Table 1 (IDT, Coralville, IA). For SYBR assays, primer specificity was evaluated with National Center for Biotechnology Information primer-BLAST (www.ncbi.nlm.nih.gov/tools/primer-blast), after which dissociation curves were generated to ensure amplification of a single gene product. All quantitative polymerase chain reaction data were normalized to glyceraldehyde 3-phosphate dehydrogenase (GAPDH) by the ΔΔC_T_ method as previously described (19).

### Protein isolation and immunoblotting

Protein homogenates were prepared with a standard radioimmunoprecipitation assay buffer supplemented by protease and phosphatase inhibitor cocktails as previously described (17). Unless otherwise specified, 45 mg of protein per well was loaded onto 12% Bis-Tris polyacrylamide gels (Invitrogen, Carlsbad, CA) and transferred to nitrocellulose membranes. Nonspecific binding was blocked with 5% nonfat dried milk suspended in Tris-buffered saline (pH 7.2) supplemented with 0.1% Tween-20 (Sigma, St. Louis, MO). Polyclonal antibody specific for sFRP4 was obtained from Abcam (Cambridge, MA) and used at 1:1000 dilution. Horseradish peroxidase–conjugated secondary antibody (Sigma-Aldrich, St. Louis, MO) was used at 1:500 dilution. Immunoreactivity was visualized with Amersham ECL Plus Detection Reagents (GE Life Sciences, Piscataway, NJ). GAPDH was used as a loading control for all experiments. Anti-GAPDH polyclonal antibody was obtained from Sigma and used at 1:1000 dilution. Results of Western blots were quantified via a LICOR Odyssey Fc Imaging system, normalized to expression of the GAPDH loading control by defining a uniform region of interest.

### Chromatin immunoprecipitation

The ChIP-IT High Sensitivity Kit (Active Motif, Carlsbad, CA) was used to crosslink and recover genomic DNA from flash-frozen tissue specimens with either a PR-specific monoclonal antibody (AB7208; Santa Cruz Biotechnologies, Dallas, TX) or a nonspecific immunoglobulin G control (AB2027; Santa Cruz). Genomic DNA corresponding to the upstream sFRP4 promoter or a noncoding region of the human genome (negative control) was quantified with SYBR Green reagents. Primers used for these analyses are described in Supplemental Table 1. PR binding was quantified after normalization to input DNA.

### Primary cell culture and biologic assays

Primary cultures of myometrium and leiomyoma were generated and maintained as previously described (20). All cultures were routinely passaged upon reaching 90% confluency. The histologic identity of these cultures was confirmed by testing for expression of smooth muscle actin and epithelial-specific markers (17). Any cultures with questionable morphology *in vitro* were routinely discarded before use.

Gene knockdown was induced by transfecting cultures at first or second passage with target-specific small interfering RNA (siRNA) or a double-stranded, nontargeting RNA control (Dharmacon, Lafayette, CO). For transfection, 1.5 × 10^5^ cells were plated in each well of a six-well plate using media supplemented with 10% fetal bovine serum but no antibiotics or antimycotic. Cultures were transfected at 70% confluence with 25-nM siRNA and Lipofectamine 2000 per well according to the manufacturer’s protocol (Life Technologies). Apoptosis was measured via Caspase 3/7 assay (Promega, Inc., Madison, WI). Proliferation was measured via an MTS assay (Cell Titer 96 Aqueous One Solution Cell Proliferation Assay, Promega). Optical density was read at 490 nm. For experiments involving steroid hormone treatment, cultures were grown to 70% confluence, after which culture media were changed to phenol red–free Dulbecco’s modiﬁed Eagle’s medium supplemented with 10% charcoal-stripped fetal bovine serum and 1% antibiotic–antimycotic. Stock solutions of estradiol (Sigma) and medroxyprogesterone (Sigma) were prepared in 100% ethanol and used to supplement individual wells to achieve doses of steroid hormone as specified. Control cultures were sham-treated with equivalent volumes of ethanol. All biologic assays were performed in triplicate.

### Measurement of secreted sFRP4

Secreted sFRP4 was measured via a customized enzyme-linked immunosorbent assay according to the manufacturer’s instructions (Wuxi Donglin Scientific Co., Wuxi/Jiangsu Province, China). All specimens were centrifuged at 1000*g* for 20 minutes at 4°C, after which the resulting supernatant was stored at −20°C. For analysis, 100 μL of media was added to each test well, with a corresponding standard dilution of bovine serum albumin, and incubated at 37°C for 2 hours. Optical density was read at 450 nm. All assays were run in duplicate.

### Immunohistochemistry

Immunohistochemical analysis of sFRP4 expression was performed with a validated tissue microarray containing >396 cores in triplicate from matched specimens of myometrium (n = 40) and leiomyomas (n = 40), smooth muscle tumors of uncertain malignant potential, and leiomyosarcoma. Antigen mobilization was performed with a standard citrate buffer (Dako, Inc., Carpinteria, CA). sFRP4-specific immunoreactivity was visualized with a rabbit polyclonal antibody at 1:12,000 dilution (Abcam). Two independent investigators (M.A.D., M.L.A.) scored expression described using a semiquantitative system that evaluated both frequency (0 to 4) and intensity of expression as previously described (17).

## Results

### Patterns of gene expression in leiomyomas and myometrium

**Figure 1.**
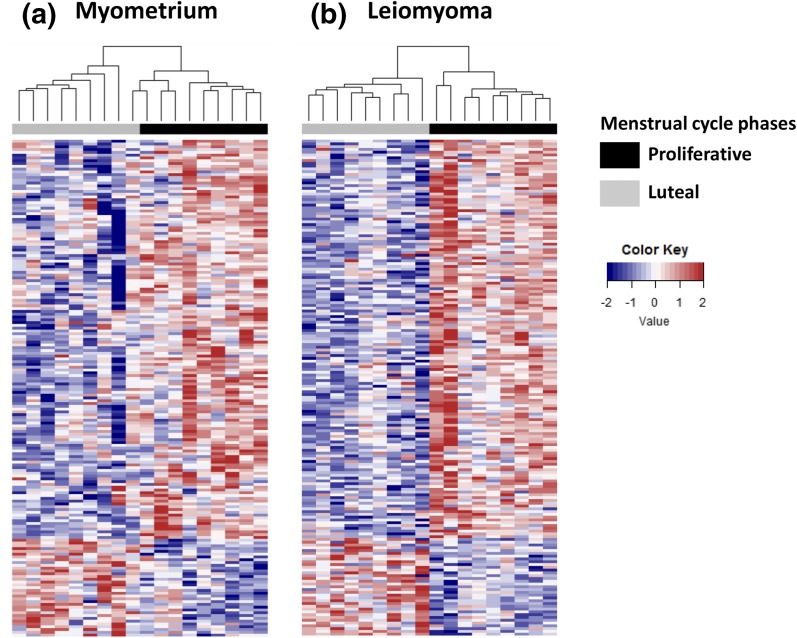
Impact of menstrual phase on patterns of gene expression in specimens of myometrium and leiomyoma. Heat maps of differentially expressed genes comparing matched specimens of (a) myometrium and (b) leiomyoma collected during the proliferative (black bar) or luteal (gray bar) menstrual phases with Euclidian hierarchical clustering (*P* < 0.01, fold change ≥ 1.5). Red, upregulated; blue, downregulated.

As a starting point to understand patterns of gene expression potentially regulated by progesterone, we comprehensively profiled and compared patterns of gene expression in matched specimens of leiomyoma and myometrium collected at distinct phases of the menstrual cycle. Recognizing the limitations inherent to the histologic criteria used to date endometrium, we screened for potential clinical outliers by examining ESR1 expression. Based on specimen clustering, we identified four outliers whose levels of ESR1 expression were inconsistent with histologic dating (Supplemental Fig. 1). Data from these four samples were removed, after which data from the remaining specimens (nine samples each of leiomyoma and myometrium, collected from proliferative or luteal phase) were used for analysis. In general, we found that changes in the patterns of gene expression that occur in myometrium or leiomyomas with progression through the menstrual cycle are highly concordant (Fig. 1).

Using two-way ANOVA analysis, we identified 178 transcripts representing 175 distinct gene products differentially expressed between specimens of leiomyoma and myometrium and between the proliferative and luteal phases of the menstrual cycle (adjusted *P* < 0.05). Of these, 143 gene products were underexpressed in luteal phase specimens, whereas 35 were overexpressed when compared with proliferative phase specimens. The 40 most differentially expressed genes in myometrium and leiomyoma are listed in Supplemental Table 2. To validate these observations, we examined levels of expression for 22 of the gene products identified by our analyses by using total RNA prepared from an additional 26 matched specimens of myometrium and leiomyoma (Supplemental Figs. 2 and 3). We specifically wanted to confirm that each gene product tested was differentially expressed in one or more phases of the menstrual cycle (proliferative, luteal, or inactive/menopausal) and whether levels of their expression were different in leiomyomas as the menstrual phase progressed. Interrogation of this second pool of specimens by RT-qPCR confirmed that each of the gene products evaluated was differentially expressed in at least one phase of the menstrual cycle when matched specimens of leiomyomas collected during the proliferative or luteal phase were compared with their matched myometrial counterparts (Supplemental Figs. 2 and 3). The only gene product whose differential expression could not be confirmed was ID1 (data not shown). We were also able to confirm that levels of DARC (*P* < 0.05), SLC2A5 (*P* < 0.05), ID3 (*P* < 0.02), LRNF5 (*P* < 0.01), UGT8 (*P* = 0.04), ESR1 (*P* < 0.01), and PGR (*P* < 0.01) were significantly lower when luteal phase leiomyomas were compared with proliferative phase leiomyomas (Supplemental Fig. 2). Lastly, we confirmed that levels of transcript encoding HES6 (*P* < 0.05), ZBTB16 (*P* < 0.02), ITPKA (*P* < 0.05), and CORIN (*P* < 0.05) were significantly higher in specimens of luteal phase leiomyomas than proliferative phase specimens (Supplemental Fig. 3). Levels of STX1A (*P* < 0.04), initially observed to increase, were significantly lower in luteal phase specimens of leiomyomas than proliferative phase specimens.

### Gene expression networks functionally linked to the PR

**Figure 2.**
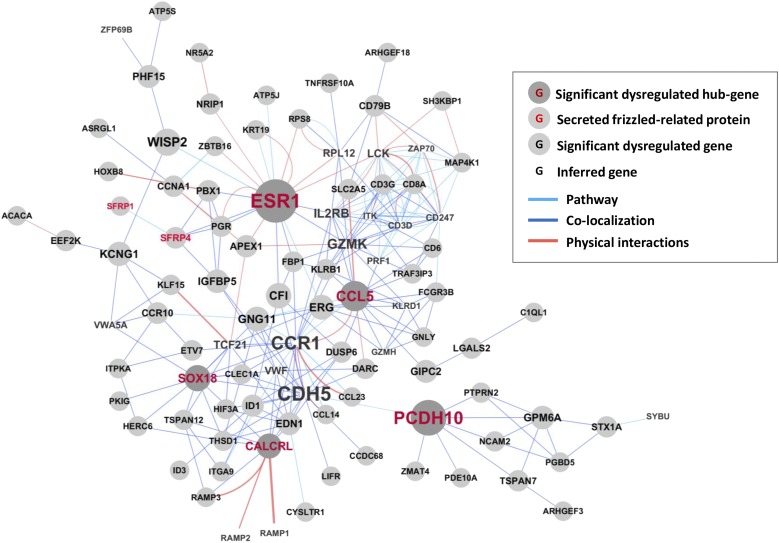
Connectivity analysis of gene products differentially expressed by tissue type and menstrual phase. Genes products identified as differentially expressed by two-way ANOVA considering both tissue type (myometrium vs leiomyoma) and menstrual phase (proliferative vs luteal) were evaluated for functional connectivity in GeneMania. Light blue bar, connectivity via recognized signaling pathway; dark blue bar, evidence of physical colocalization; orange bar, known physical interactions. Font size for each gene product identified is proportional to connectivity. Presence of a gray circle in the background indicates overexpression or underexpression when specimens of leiomyomas were compared with healthy myometrium. The five most connected hubs are indicated by gene symbols in red type. Members of the sFRP family of Wnt inhibitors are indicated by gene symbols in orange type. Gene products inferred to be important for function of observed networks are denoted by their gene symbol alone. No evidence of differential expression was observed for this latter category of genes.

Next, we undertook an analysis of our transcriptional profiling results by using GeneMANIA (21). GeneMANIA is an established bioinformatic tool that integrates large amounts of data ranging from gene–gene interactions to gene expression data sets to parse and prioritize functional associations between sets of input genes and find additional genes and pathways relevant to loci identified by genetic screens. Application of this algorithm to our data revealed that the differentially expressed genes in leiomyoma affected by menstrual phase are functionally organized around multiple hubs (Fig. 2). When ranked by betweenness centrality and number of neighbors in their network (see also Supplemental Table 3), these hubs, in decreasing magnitude of connectivity, are the ESR1, SOX18, CCL5, CALCRL, and PCDH10. This result suggests that activity of these gene products plays a critical role in mediating the role of progesterone in uterine smooth muscle tumorigenesis. As a starting point to explore this observation, we used RT-qPCR to examine expression of these genes and verify that they are differentially expressed in leiomyomas and that levels of their expression vary by menstrual phase. Using total RNA prepared from our second panel of 26 matched specimens, we confirmed that ESR1, SOX18, CCL5, and PCDH10 but not CALCRL are differentially expressed in uterine leiomyomas or that levels of their expression vary by menstrual phase (Supplemental Figs. 2 and 4).

**Figure 3.**
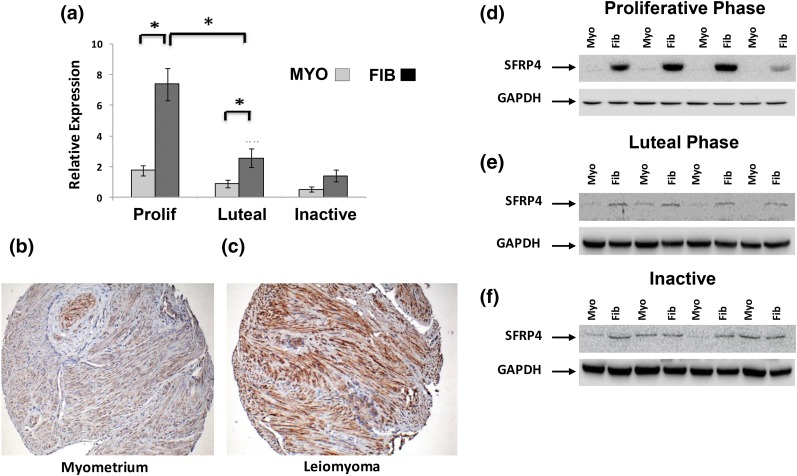
Characterization of sFRP4 expression in specimens of myometrium and leiomyoma. (a) Relative expression of sFRP4 transcript evaluated by RT-qPCR with total RNA prepared from specimens of myometrium (MYO) and leiomyoma (FIB) collected from the proliferative (Prolif) (n = 10), luteal (n = 9), or menopausal (inactive) women (n = 7). All results are reported as relative expression normalized to GAPDH expression with standard errors of the mean (*P < 0.01). Expression of sFRP4 protein was also evaluated by Western blot with total protein prepared from matched specimens of myometrium (Myo) and leiomyomas (Fib) collected during (d) proliferative or (e) luteal phase of the menstrual cycle or from (f) postmenopausal women (inactive). Immunohistochemical evaluation of sFPR4 expression in representative luteal phase specimens of (b) myometrium and (c) leiomyoma. All images at ×40 magnification.

Our analyses indicated that among genes differentially expressed in leiomyomas, the PR is functionally linked to both ESR1 and sFRP4. In our initial whole genome transcriptional profiling, sFRP4 expression was both 2.6 times greater in fibroids as compared with myometrium (*P* < 0.01) and 2.5 times greater in leiomyomas collected in the proliferative phase than specimens collected during the luteal phase (*P* < 0.01). Examining our additional 26 matched specimens of myometrium and leiomyoma, we found that levels of sFRP4 transcript were 4.2 times higher in leiomyomas collected during the proliferative phase of the menstrual cycle (n = 10, *P* < 0.001) but only 2.9 times higher in specimens of luteal phase leiomyomas (n = 9, *P* < 0.01) [Fig. 3(a)]. No difference in levels of sFRP4 transcript were observed when specimens of leiomyoma and myometrium from postmenopausal women (n = 7) were compared.

A similar pattern of sFRP4 expression was observed when protein lysates prepared from the same specimens were evaluated by Western blot [Fig. 3(d), 3(e), and 3(f)]. We also evaluated sFRP4 expression by immunohistochemistry by using a tissue microarray constructed from a third set of matched leiomyoma and myometrial specimens. As shown in Fig. 3(b) and 3(c), sFRP4-specific immunoreactivity was granular and primarily localized to the perinuclear region of uterine smooth muscle cells. Semiquantitative assessment of sFRP4 expression confirmed that sFRP4 is robustly overexpressed in specimens of leiomyomas collected from the proliferative and luteal phase of the menstrual cycle (n = 20 each group; data not shown). Although the mean expression score for sFRP4 in luteal leiomyomas was lower than in proliferative phase specimens, this difference was not statistically significant. No qualitative differences patterns of sFRP4 immunoreactivity were observed when proliferative and luteal phase specimens of myometrium and leiomyomas were compared (data not shown).

### sFRP4 expression in primary uterine smooth muscle culture

**Figure 4.**
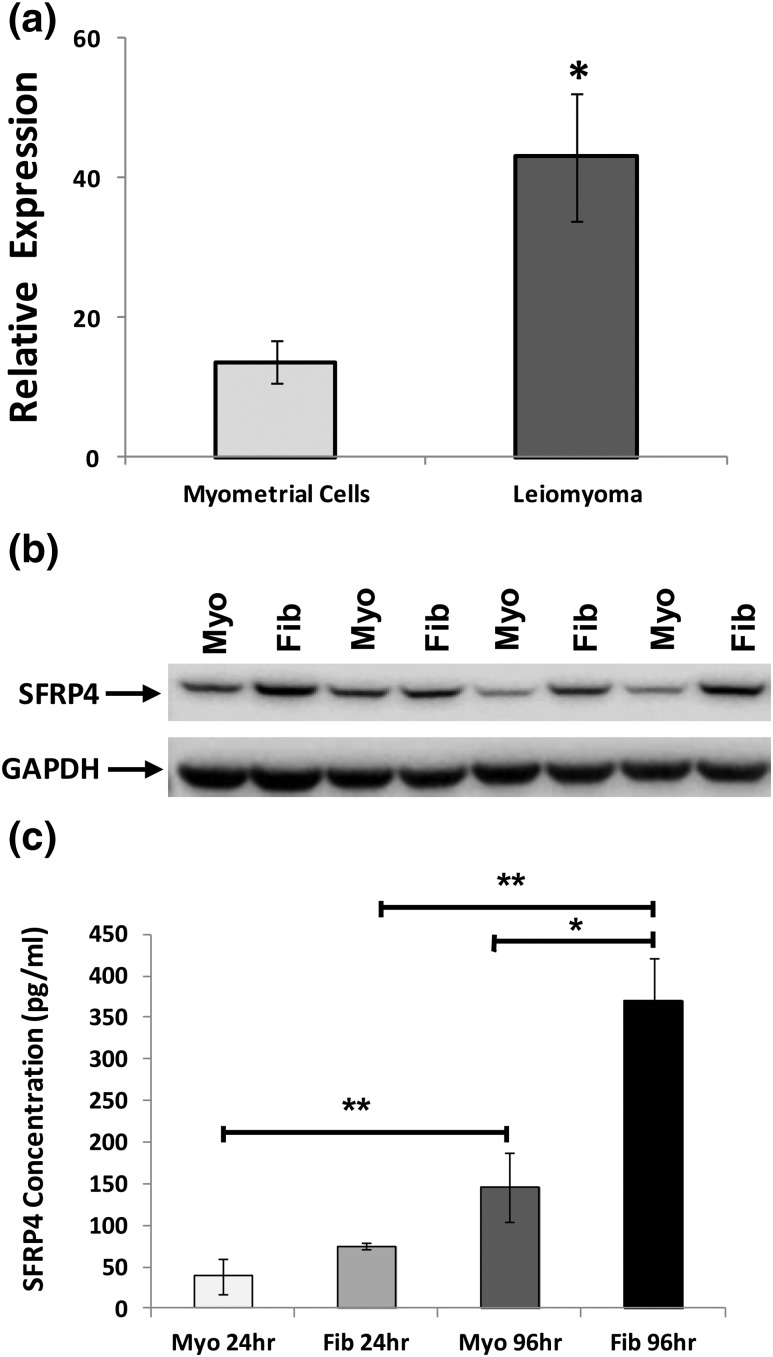
Characterization of sFRP4 expression in primary cultures derived from specimens of myometrium and leiomyoma. (a) Expression of sFRP4 transcript evaluated with RT-qPCR, with results reported as relative fold-levels of expression with standard errors of the mean. (b) sFRP4 expression evaluated in specimens of myometrium (Myo) and leiomyoma (Fib) by Western blot. (c) Evaluation of spent media for secreted sFRP4 by enzyme-linked immunosorbent assay. Media were aspirated from primary cultures of either myometrium or leiomyomas after 24 or 96 hours. (*P ≤ 0.001; **P ≤ 0.01).

To determine whether overexpression of sFPR4 observed in leiomyomas persists *in vitro*, we compared levels of this gene product in primary cultures derived from myometrium and leiomyoma. We found that levels of sFRP4 messenger RNA and protein were significantly higher in cultures derived from leiomyomas than from myometrium, as measured by quantitative polymerase chain reaction [Fig. 4(a)] and Western blot [Fig. 4(b)]. High levels of sFRP4 expression and its differential expression persisted in cultures derived from these specimens for at least three passages. Of note, levels of sFRP4 increased with confluency, regardless of whether that culture had been obtained from a specimen of myometrium or leiomyoma (data not shown). In other cells and tissues, sFRP4 functions as a secreted, paracrine inhibitor of Wnt signaling. Therefore, we also sought to determine whether primary cultures of myometrium and leiomyomas secrete sFRP4 into their local environment. As shown in Fig. 4(c), sFRP4 was readily detected in media from cultures of both myometrium and leiomyoma and that significantly more sFRP4 was secreted over time from primary cultures of leiomyoma.

### Regulation of sFRP4 expression by progesterone

**Figure 5.**
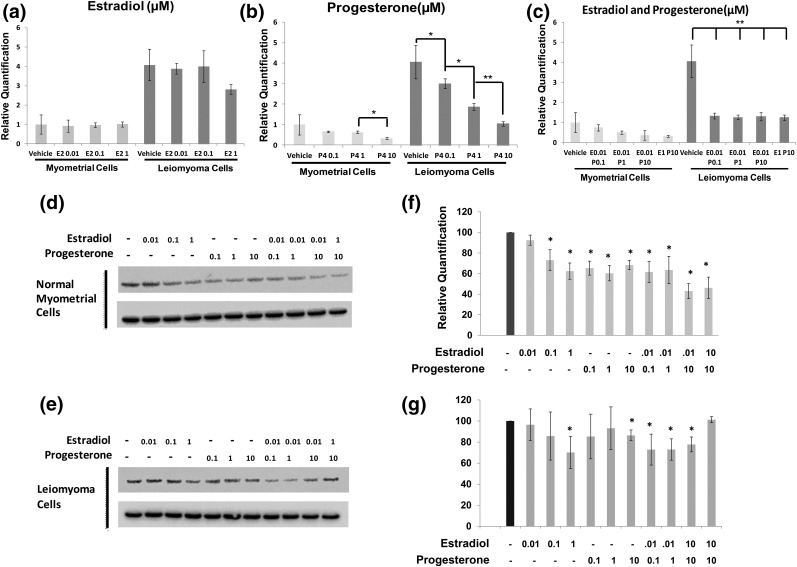
Impact of estrogen and progesterone treatment on sFRP4 expression *in vitro*. (a–c) Primary cultures derived from myometrium or leiomyoma were incubated with vehicle alone or doses of estradiol (E2), progesterone (P4), or combinations of estrogen and progesterone across a range of doses as specified. Expression of sFRP4 transcript, normalized to GAPDH, was then evaluated by RT-qPCR. Results are reported as relative fold-levels of expression with standard errors of the mean (*P = 0.05; **P < 0.01). sFRP4 expression was also evaluated in primary cultures (n = 3) of (d) myometrium and (e) leiomyoma cells after treatment with media supplemented with estradiol, progesterone, or estradiol plus progesterone by Western blot. Expression of GAPDH was used as a loading control. (f, g) Relative quantification of Western blots performed to evaluate impact of increasing micromolar concentrations of estradiol and progesterone on sFRP4 expression in primary cultures of myometrium (n = 3) and leiomyoma (n = 3) (*P < 0.05 when compared with cultures treated with vehicle alone). For all panels, doses of estradiol and progesterone are specified as micromolar (μM) concentrations at final dilution.

We wanted to explore the functional relationship between ER, PR, and sFRP4. We identified a single PR consensus binding site starting 293 bases upstream from the transcriptional start site for the sFRP4 gene (data not shown). No consensus binding sites for the ER were identified. Evaluation of genomic DNA immunoprecipitated by a high-fidelity antibody specific to the PR demonstrated clear enrichment for sFRP4 promoter in specimens of both myometrium and leiomyoma as compared with control input DNA precipitated with nonspecific immunoglobulins (Supplemental Fig. 5). To evaluate the functional impact of PR binding to this site, we treated primary cultures of leiomyoma and myometrium maintained in serum-free and phenol red–free media with different doses and combinations of estradiol and progesterone. We found that the treatment of primary cultures derived from premenopausal leiomyoma (n = 3) with progesterone reduced levels of sFRP4 transcript in a dose-dependent fashion [Fig. 5(b) and 5(e)]. Decreased expression of sFRP4 transcript was also noted in primary cultures of myometrium treated at high concentrations of progesterone [Fig. 5(b)]. As predicted by the lack of an estradiol binding site in the sFRP4 promoter, no significant changes in the levels of sFRP4 transcript were observed in response to estradiol alone [Fig. 5(a)]. Lastly, we found that low doses of estradiol (0.01 μM) significantly potentiated the ability of progesterone to inhibit expression of sFRP4 transcript in leiomyomas [Fig. 5(c)].

Next, we examined the impact on estradiol and progesterone treatment on the expression of sFRP4 protein. Consistent with our RT-qPCR results, we found that levels of sFRP4 protein in primary cultures of leiomyoma were remarkably sensitive to progesterone at all doses tested [Fig. 5(d) and 5(f)]. We also found that levels of sFRP4 protein were reduced in a dose-dependent fashion by progressively increasing concentrations of estradiol. However, estradiol failed to potentiate the ability of progesterone to inhibit sFRP4 expression myometrial cells at either dose tested [Fig. 5(d) and 5(f)]. In contrast, cultures of leiomyoma appeared less responsive to both estradiol and progesterone. As shown in Fig. 5(e) and 5(g), decreased expression of sFRP4 protein was observed only at high (1 μM) concentrations of progesterone or estradiol. However, low doses of estradiol did potentiate progesterone’s ability to inhibit sFRP4 expression in primary leiomyoma cultures.

### Functional role of sFRP4 in uterine smooth muscle

**Figure 6.**
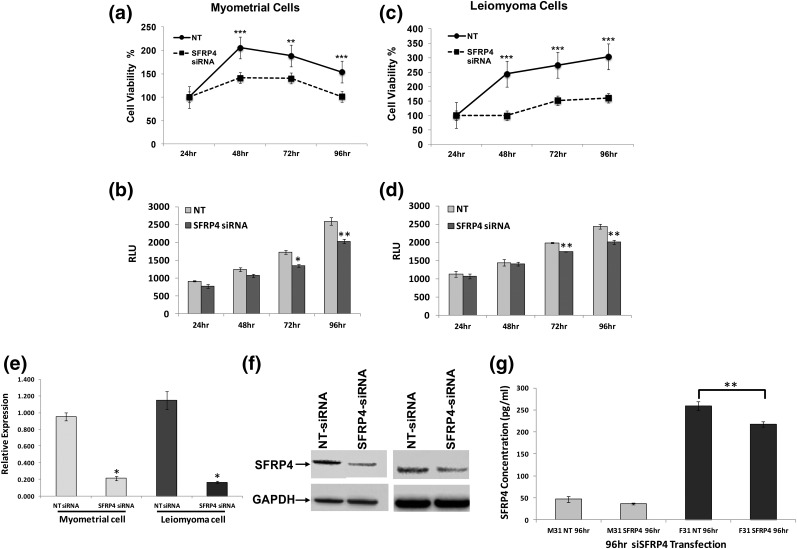
Impact of targeting sFRP4 expression on primary cultures of myometrium and leiomyoma. Primary cultures derived from (a, b) myometrium or (c, d) leiomyoma were transfected with either siRNAs specific to sFRP4 (sisFRP4) or a nontargeting (NT) control, after which (a, c) proliferation and (b, d) apoptosis were evaluated as described (*P < 0.05; **P < 0.02; ***P < 0.01). Results of each assay were standardized to the initial measurement made at 24 hours. Levels of sFRP4 in transfected cultures were evaluated by (e) RT-qPCR and (f) Western blot. For RT-qPCR, results are reported as relative fold-increases in expression with standard errors of mean. For Western blots, expression of GAPDH was used as a loading control (*P ≤ 0.001). (g) sFRP4 measured in spent media from primary cultures transfected with siRNA targeting sFRP4 or an NT control (n = 3; **P < 0.05). For all panels, time is specified in hours. F, leiomyoma cells; M, myometrial cells; RLU, relative luminescence units.

Given its well-recognized role as a secreted Wnt inhibitor, we hypothesized that sFRP4 plays a critical role of regulating rates proliferation and apoptosis in uterine smooth muscle. As shown in Fig. 6, knockdown of sFRP4 resulted in decreased rates of proliferation and apoptosis in primary cultures of both leiomyoma and myometrium. We also found that levels of cumulative sFRP4 secretion measured in ambient culture media were reduced when primary leiomyoma cultures were transfected with double-stranded siRNA targeting its intracellular expression [Fig. 6(g)].

## Discussion

Dissecting the mechanisms by which activation of the PR promotes leiomyoma growth will ultimately help develop more effective strategies for treating these tumors. Our observations clearly demonstrate that overexpression of sFRP4 is a robust, progesterone-regulated feature of uterine leiomyomas. Encoded at 7p14.1, sFRP4 is a secreted glycoprotein whose expression has been implicated in multiple physiologic processes, including embryonic development, angiogenesis, and apoptosis (22–24). High serum levels of sFRP4 have also been previously reported in multiple pathologic conditions, including obesity and osteoporosis (25–27). Elevated circulating levels of sFRP4 have also been observed several years before a diagnosis of type 2 diabetes, where it may play a critical role in linking islet inflammation with impaired insulin secretion (28).

To the best of our knowledge, the overexpression of sFRP4 in uterine leiomyomas has not been previously described, nor has sFRP4 been specifically reported to be a progesterone-responsive gene. At present, the mechanisms driving the high levels of sFRP4 expression in leiomyomas are not known. However, our data indicate that progesterone probably inhibits sFRP4 expression by directly binding to a previously unappreciated consensus binding site in its upstream promoter. Surprisingly, we also found that estradiol is able to inhibit the expression of sFRP4 protein (Fig. 5), despite the lack of an upstream consensus binding site for ESR1 in the sFRP4 promoter. Given that levels of sFRP4 transcript were not significantly affected by the application of estradiol to primary cultures of myometrium or leiomyoma *in vitro*, it is possible that the ability of estradiol to inhibit sFRP4 expression ultimately relies on a posttranscriptional mechanism. However, it is also possible that our observations are explained by the well-recognized ability of estrogen to increase PR expression. This latter hypothesis is consistent with the fact that low doses of estradiol potentiated the response of leiomyomas to progesterone *in vitro* (Fig. 5). We are not currently able to distinguish which of these possible mechanisms is responsible for our observations.

Another key question that arises from our work is how inhibition of high levels of sFRP4 expression (Figs. 3–5) in leiomyomas by activation of the PR contributes to tumor growth during the luteal menstrual phase. In other cells and tissues, members of the sFRP family function by directly binding Wnt ligands and sequestering them from active receptor complexes. sFRP4-induced signaling events are capable of inhibiting both canonical and noncanonical Wnt signaling (29). In mice, constitutive overexpression of activated *β*-catenin, a key effector of canonical Wnt pathways, in uterine mesenchyme leads to leiomyoma-like proliferations at high penetrance (30). Small-molecule inhibitors for at least three different canonical Wnt pathway effectors have also been shown to exert antiproliferative effects on primary cultures of leiomyoma cells *in vitro* (31). In contrast, our data clearly demonstrate that knockdown of sFRP4 inhibits the proliferation of leiomyoma cells *in vitro* (Fig. 6). These observations suggest that sFRP4 overexpression promotes leiomyoma growth. In part, our observations *in vitro* probably reflect the culture conditions used to perform these experiments. *In vivo*, uterine leiomyomas are characterized by a dense extracellular matrix that plays an important role in determining key cellular functions (32). It is possible that, when removed from this context, leiomyoma cells respond differently to sFRP4. Consistent with this hypothesis, *SFRP4* has been previously shown to encode a netrin-related motif at its carboxy terminus. A similar motif is found in other gene products typically found in tumor microenvironments, including tissue inhibitors of metalloproteinases, type-1 procollagen C-proteinase enhancer proteins, and complement proteins (33). Additional work will be needed to confirm that sFRP4 promotes leiomyoma growth *in vivo*.

In part, the mechanisms by which the tight regulation of sFRP4 expression in uterine leiomyomas promotes their growth may depend on the subpopulation of cells within the tumor complex on which sFRP4 exerts its impact. sFRP4 has been previously shown to play a key role in regulating mesenchymal stem cell populations in adipose and other tissues (34, 35). Side populations capable of repopulating tumors *in vivo* have also been recently been recovered from both healthy human myometrium and uterine leiomyomas (36). These cells, which express ER and PR only at low levels, are critical for leiomyoma growth *in vivo* (37, 38). It has been proposed that canonical Wnt pathways are activated in myometrial stem cells by the expression of WNT11 and WNT16 induced by steroid hormone exposure in differentiated myometrial cells (15). Furthermore, inhibition of *β*-catenin and T-cell factor 4, two key effects of Wnt signaling, have been previously shown to suppress the ability of leiomyoma side populations to populate tumors *in vivo*. In this context, our data suggest that another potentially important mechanism by which progesterone contributes leiomyoma growth is by reducing antagonism to Wnt signaling in the microenvironment of tissue-specific stem cells.

## Conclusions

We believe that our observations identify an important mechanism mediating the impact of progesterone on uterine leiomyomas. Additional work is needed to elucidate how the overexpression of sFRP4 contributes to the ability of progesterone to promote leiomyoma growth and determine how its distinct pattern of regulation contributes to uterine smooth muscle tumorigenesis. Long-term efforts to better understand these mechanisms will help determine whether or how small-molecule inhibitors for sFRP4 could be used to control symptoms of leiomyomas.
