# Supplementary material for: A Role for Progesterone-Regulated sFRP4 Expression in Uterine Leiomyomas
Source: J Clin Endocrinol Metab. 2017 Jun 14;102(9):3316–26. doi: 10.1210/jc.2016-4014 (PMC5587057; doi:10.1210/jc.2016-4014)
Supplement: Supplementary file 1 [file jc.2016-4014.sd1.pdf]

<u>Figure S1.</u> Unsupervised K-mean clustering on ESR1 expression reveals four outlier samples.

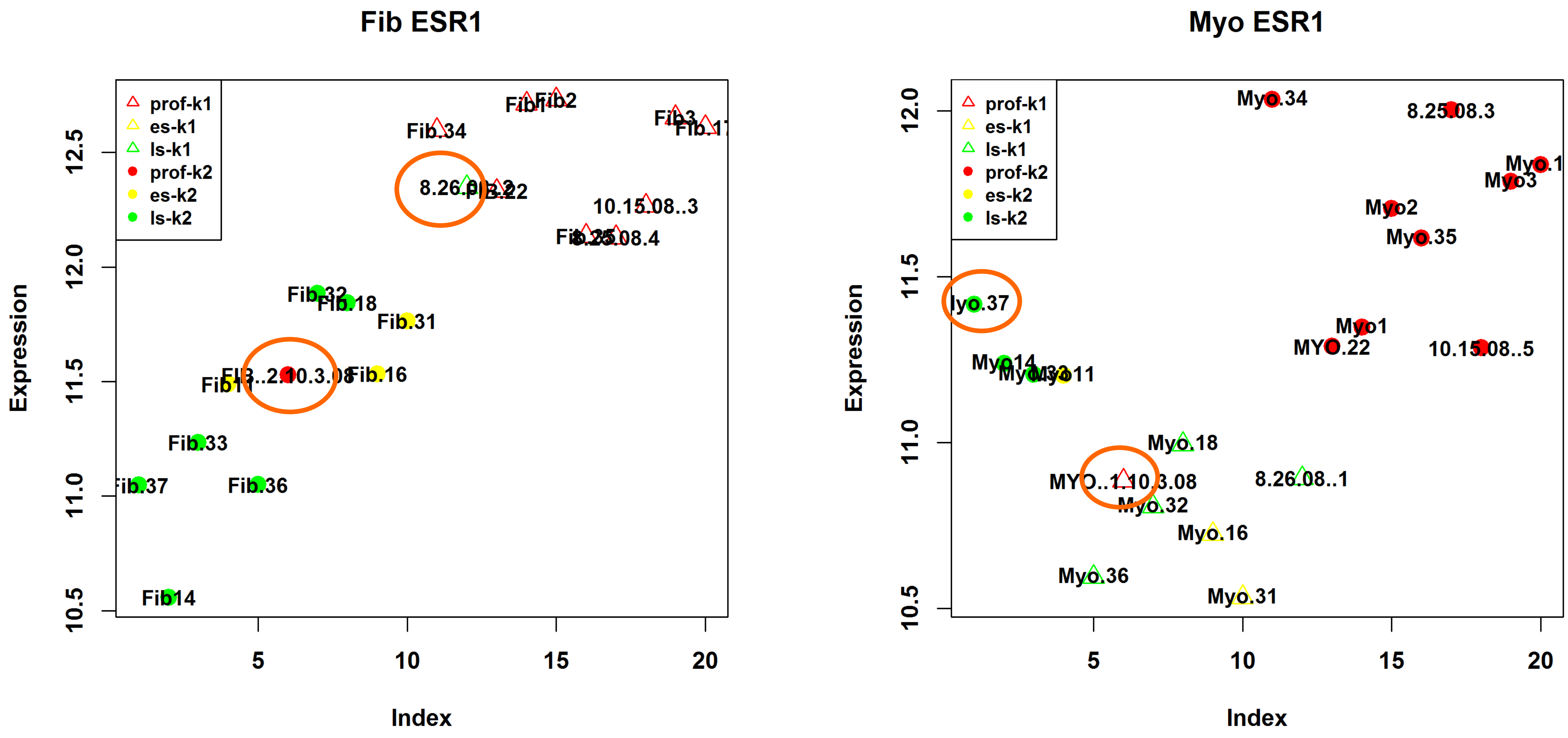

- 10.3.08 (proliferative) always low as secretory in both tissues.
- 8.26.08 (late secretory) high as proliferative in fibroid (leiomyoma).
- Myo.37 (late secretory) high as proliferative in myometrium.

Figure S2. Differentially expressed gene products whose levels were initally found to decrease in leiomyomas with progression through the menstrual cycle.

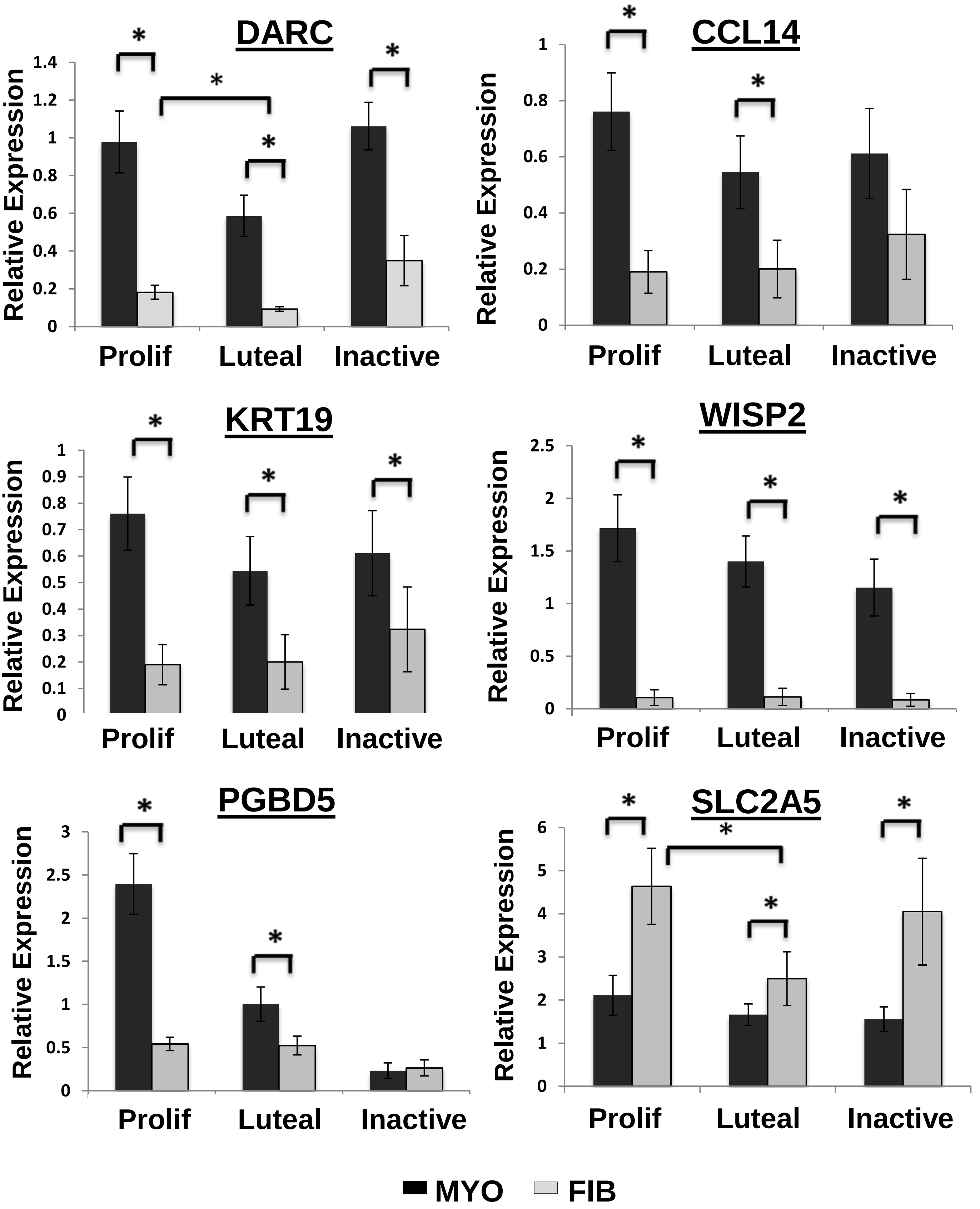

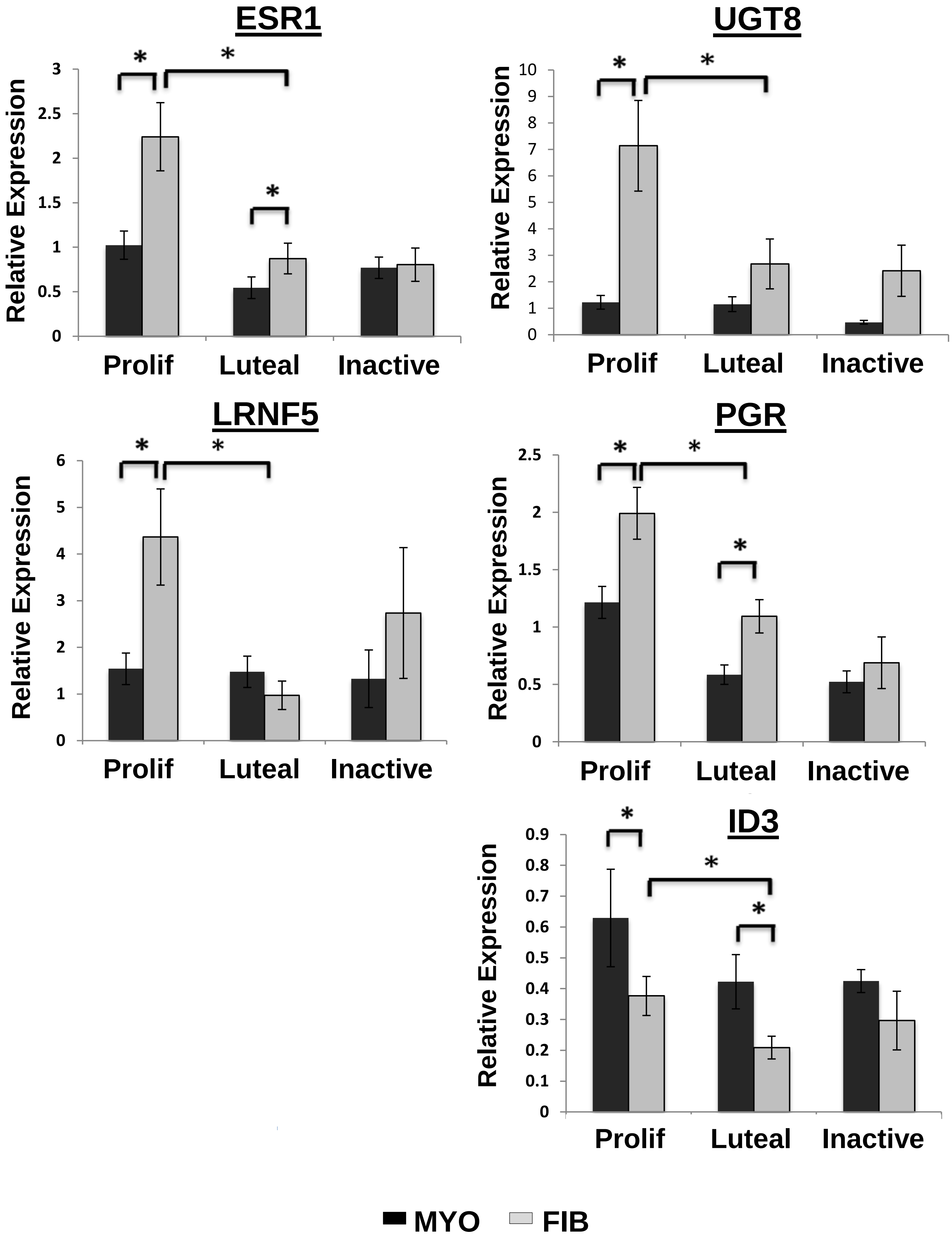
ESR1
UGT8
LRNF5
PGR
ID3
Relative Expression
Prolif
Luteal
Inactive
*
MYO
FIB

Figure S3: Differentially expressed gene products whose levels were initially found to increase in leiomyomas with progression through the menstrual phase.

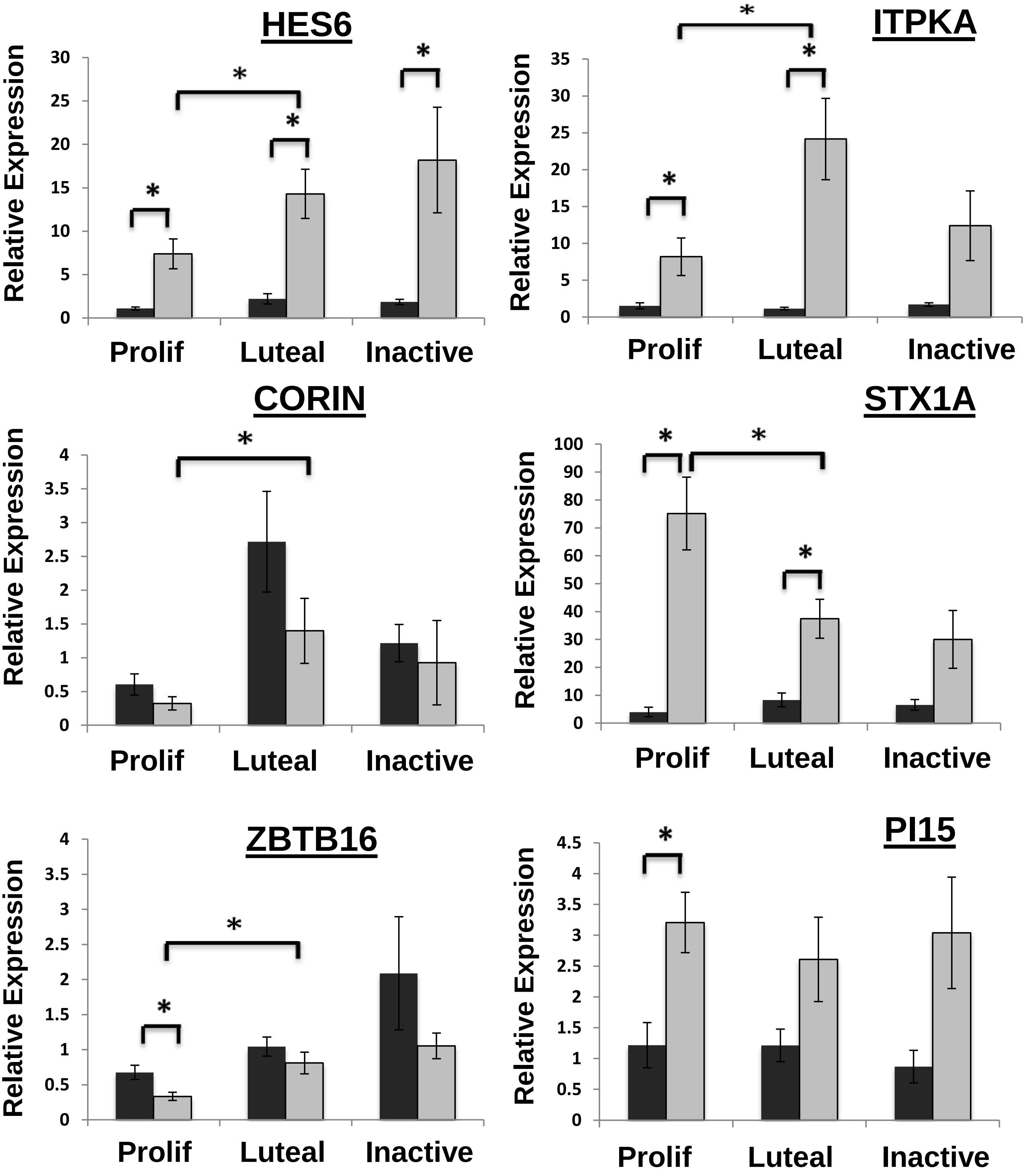

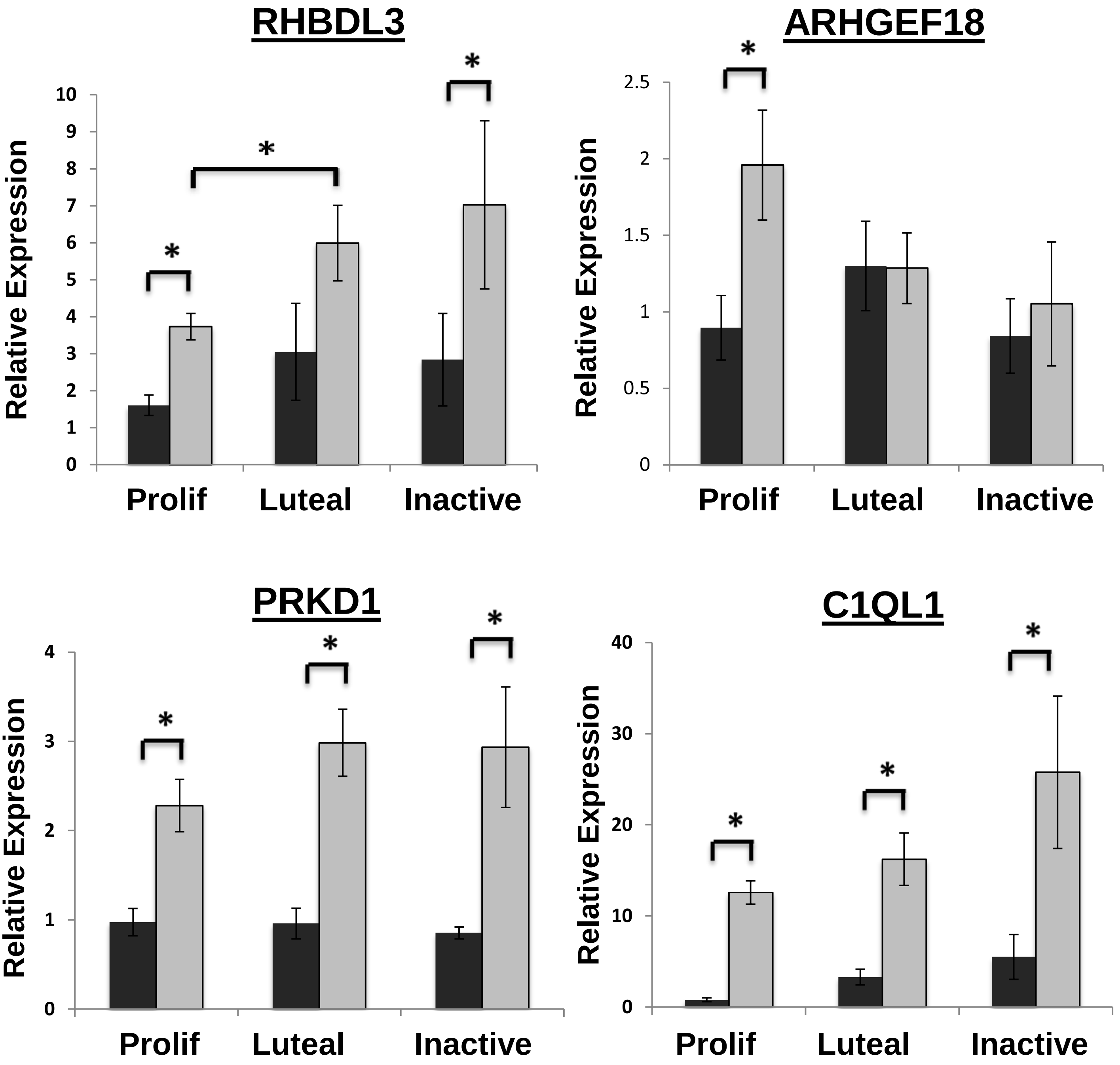
RHBDL3
Relative Expression
0
1
2
3
4
5
6
7
8
9
10
*
*
*
*
Prolif
Luteal
Inactive
ARHGEF18
Relative Expression
0
0.5
1
1.5
2
2.5
*
Prolif
Luteal
Inactive
PRKD1
Relative Expression
0
1
2
3
4
*
*
*
Prolif
Luteal
Inactive
C1QL1
Relative Expression
0
10
20
30
40
*
*
*
Prolif
Luteal
Inactive

Figure S4. Relative expression of gene products identified as key progesterone-regulated "hubs" by GeneMania analysis

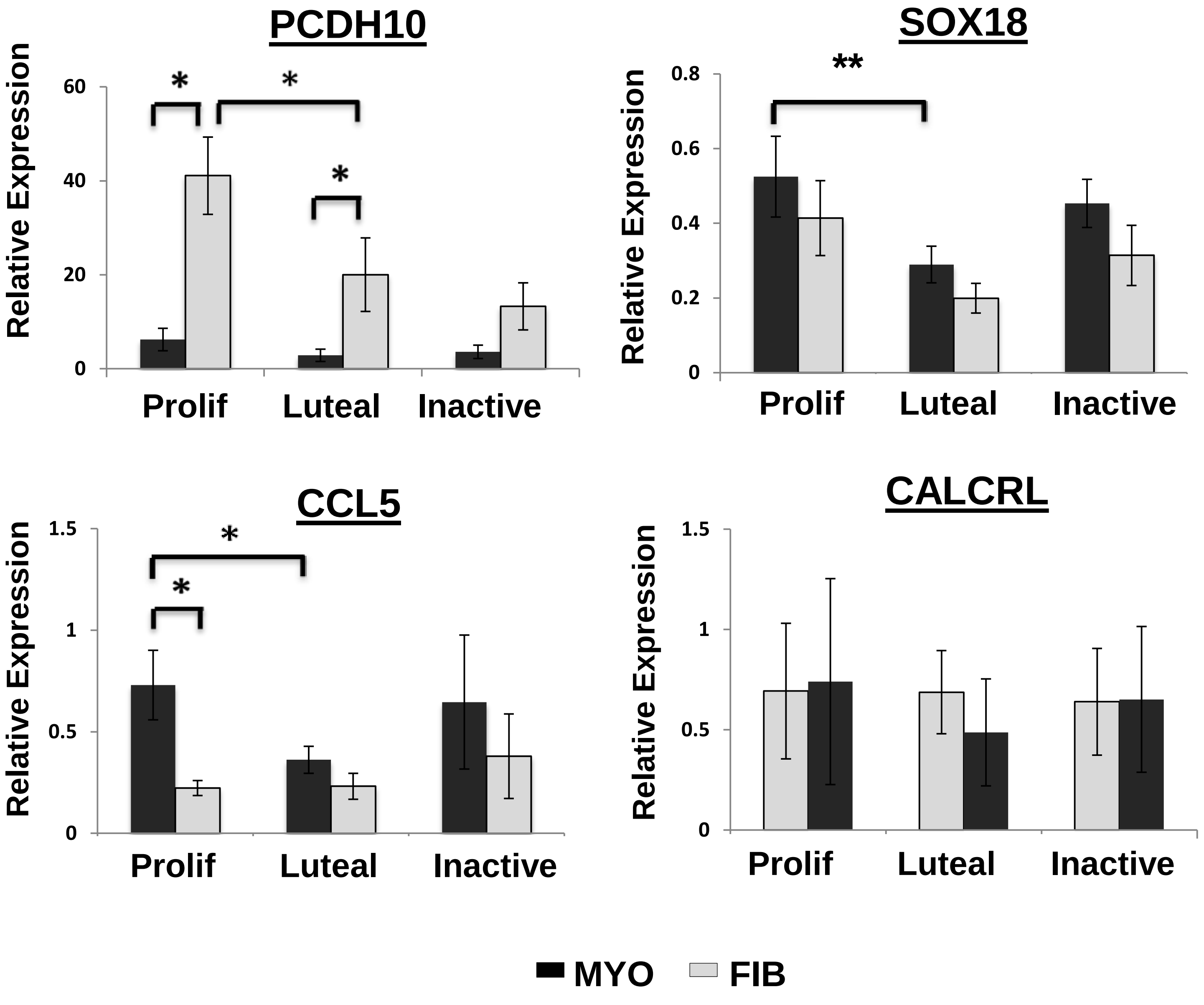

Figure S5.

G.

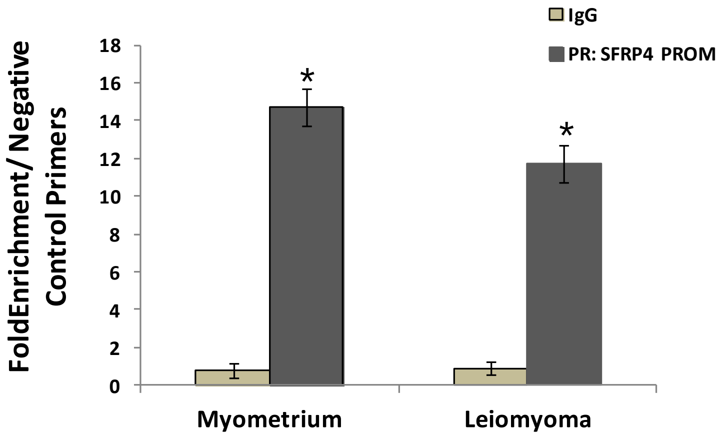

IgG
PR: SFRP4 PROM
FoldEnrichment/ Negative Control Primers
18
16
14
12
10
8
6
4
2
0
*
*
Myometrium
Leiomyoma

Table S1. Primers used to evaluate the expression of putative progesterone-regulated gene products.

Gene	Forward Primer (5'->3')	Reverse Primer (5'->3')
ITPKA	AGCACCGACTTCAAGACTACGC	CCTCCTGAAGAACTCGGATACC
STX1A	TGGAGAACAGCATCCGTGAGCT	CCTCTCCACATAGTCTACCGCG
ARHGEF18	TTCAGCCTGCCCGAAGAGGAAA	TCTAGGAGGCTCTGTGCGATCA
PRKD1	GAGGACGCCAACAGAACCATCA	CTGTGCTGCTTTTCCTCTTCGTG
C1QL1	AGTATGTGGGCAGACCTCTGCA	CCAGCTTGATGAAGACCTCGTC
TMEPA1	CTGAGCCACTACAAGCTGTCTG	GGATTCCGTTGCCTGACACTGT
PI15	TCGCAGAATGACATGATCGC	TGGTCCCAAATGCAAGTAGC
UGT8	GGAGGAATCCTAACCAAACCAG	TCCTGCCAGTTTGTTAGCAATGT
ESR1	GCTTACTGACCAACCTGGCAGA	GGATCTCTAGCCAGGCACATTC
SFRP5	CTGAGATGCTGCACTGCCACAA	GTCAGCACTGTGCTCCATCTCA
DARC	GGGCTGAAGAAGGCATTGGGTA	CTTGGACCTCACCAGGAAATCC
CCL14	TGATGTCAAAGCTTCCACTGGAA	GAGTGAACACGGGATGCTTTGT
KRT19	ATGCAGATCGAAGGCCTGAA	TTGGCTTCGCATGTCACTCA
WISP2	CCTGCGACCAACTCCACGTCT	TTCACCTCACAGCTGCTGTCGT
SLC2A5	GGCTTCTCCATCTGCCTCATAG	GGAGATGACACAGACGATGCTG
ID3	CAGCTTAGCCAGGTGGAAATCC	GTCGTTGGAGATGACAAGTTCC
LRFN5	GCAGAAGCTACATCATCAACGG	GACCAGAAAAGTTTTGCTCGTAG
ID1	GTTGGAGCTGAACTCGGAATCC	ACACAAGATGCGATCGTCCGCA
CPXM2	CAGAGGATCGACAGAATGTCCC	CATCCAGGCTATGACTGCTCTG
PGBD5	CAAACCCAGGTGCTACATGA	GCTTATTCTTCAGCGCATCC
CORIN	GTTCCCAGACTGCCCTGATTAC	ACTGTCTGAGCAGTCGGCTTCA
ZBTB16	GAGCTTCCTGATAACGAGGCTG	AGCCGCAAACTATCCAGGAACC
RHBDL3	GAACACCTGGGACTCAATGTGG	TGTCAGCCACAGACACTGCCAA
SFRP4 Promoter	CACCTTCCCCCAGTTCTGTT	CACTCCGCCCTTAGTTGTGT
SFRP4	AGTTCCTGCACGACCCTATCA	TAACATCCTCCGGGAGGTCC

Table S2.

Top 20 Under-Expressed Genes: Leiomyomas in Luteal Phase

Gene ID	Entrez ID	Names and Symbols	Functions	Fold Change	P Value
DARC	2532	Duffy Blood Group, Atypical Chemokine Receptor	Glycosylated membrane protein,non-specific receptor for several chemokines	-3.55	0.018
CCL14	6358	Chemokine (C-C Motif) Ligand 14	Induces changes in intracellular Ca2+ concentration and enzyme release in monocytes	-2.82	0.008
KRT19	3880	Keratin 19	Involved in organization of myofibers. Together with KRT8, helps to link the contractile apparatus to dystrophin at the costameres of striated muscle	-2.65	0.031
WISP2	8839	WNT1 Inducible Signaling Pathway Protein 2	Member of the WNT1 inducible signaling pathway (WISP)subfamily; modulation of bone turnover	-2.57	0.018
PGBD5	79605	PiggyBac Transposable Element Derived 5	Novel transposase	-2.56	0.0002
**SFRP4**	6424	Secreted Frizzled-Related Protein 4	Acts as a soluble modulator of Wnt signaling pathway; regulation of cell growth and differentiation	-2.48	0.012
SLC2A5	6518	Solute Carrier Family 2 (Facilitated Glucose/Fructose Transporter), Member 5	Primarily a fructose transporter; Cytochalasin B-sensitive carrier	-2.34	0.001
ID3	3399	Inhibitor Of DNA Binding 3, Dominant Negative Helix-Loop-Helix Protein	Forms heterodimers with other HLH proteins,inhibiting DNA binding. Involved in Myogenesis: promoting muscle precursor cells proliferation	-2.31	0.004
LRFN5	14558	Leucine Rich Repeat And Fibronectin Type III Domain Containing 5	Cell adhesion molecule that mediates homophilic cell-cell adhesion in a Ca(2+)-independent manner	-2.22	0.006
ID1	3397	Inhibitor Of DNA Binding 1, Dominant Negative Helix-Loop-Helix Protein	Forms heterodimers with other HLH proteins,inhibiting DNA binding; endometrial carcinoma	-2.18	0.028
CPXM2	119587	Carboxypeptidase X (M14 Family), Member 2	Cell-cell interactions	-2.16	0.034
UGT8	7368	UDP Glycosyltransferase 8	Catalyzes key step in the biosynthesis of galactocerebrosides (sphingolipids of the myelin membrane of the CNS and PNS)	-2.08	0.030
ESR1	2099	Estrogen Receptor 1	Nuclear hormone receptor. Involved in the regulation of gene expression, affecting cellular proliferation and differentiation in target tissues. Essential for reproductive function.	-2.07	5.855 E-05
CCNA1	8900	Cyclin A1	Cyclins function as regulators of CDK kinases; functions in the control of the germline meiotic cell cycle	-2.04	0.037
GYPC	2995	Glycophorin C (Gerbich Blood Group)	Integral membrane glycoprotein. Minor species carried by human erythrocytes, regulating the mechanical stability of red cells.	-2.04	0.014
SOX17	64321	SRY (Sex Determining Region Y)-Box 17	Member of SOX (SRY-related HMG-box) family of transcription factors;regulation of embryonic development,determines the cell fate.	-2.00	0.016
PGR	5241	Progesterone Receptor	Mediates physiological effects of progesterone; central role in reproductive events:establishment and maintenance of pregnancy	-1.99	0.0003
LFNG	3955	LFNG O-Fucosylpeptide 3-Beta-N-Acetylglucosaminyltransferase	Glycosyltransferases that act in the Notch signaling pathway to define boundaries during embryonic development	-1.98	0.023
CYSLTR1	10800	Cysteinyl Leukotriene Receptor 1	Receptor for cysteinyl leukotrienes mediating bronchoconstriction	-1.97	0.0003
OR2A9P	441295	Olfactory Receptor, Family 2, Subfamily A, Member 9 Pseudogene	Responsible for the recognition and G protein-mediated transduction of odorant signals.	-1.94	0.014

Top 20 Over-Expressed Genes: Leiomyomas in Luteal Phase

Gene ID	Entrez ID	Names and Symbols	Functions	Fold Change	P Value
Hes 6	55502	Hes Family BHLH Transcription Factor 6	Transcription repressor; regulation of cell differentiation	3.01	0.008
ITPKA	3706	Inositol-Trisphosphate 3-Kinase A	Regulates inositol phosphate metabolism (impt for cell signaling)	2.82	0.031
CORIN	10699	Corin, Serine Peptidase	Converts pro-atrial natriuretic peptide to biologically active atrial natriuretic peptide	2.70	0.049
STX1A	6804	Syntaxin 1A (Brain)	Docking of synaptic vesicles with the presynaptic plasma membrane	2.23	0.044
ZBTB16	7704	Zinc Finger And BTB Domain Containing 16	Cell cycle progression, interacts with a histone deacetylase	2.09	0.013
PI15	51050	Peptidase Inhibitor 15	Trypsin inhibitor, possible role in central nervous system	2.07	0.020
PRKD1	5587	Protein Kinase D1	Serine/threonine kinase; membrane receptor signaling, transport at the Golgi, protection from oxidative stress,regulation of cell motility and adhesion	2.06	0.027
C1QL1	10882	Complement Component 1, Q Subcomponent-Like 1	May regulate the number of excitatory synapses formed on neurons	1.96	0.009
RHBDL3	2E+05	Rhomboid, Veinlet-Like 3	Involved in intramembrane proteolysis and subsequent release of functional polypeptides from their membrane anchors	1.91	0.045
RHGEF1	23370	Rho/Rac Guanine Nucleotide Exchange Factor (GEF) 18	Cytoskeletal rearrangements, gene transcription, cell growth and motility	1.75	0.009
EEF2K	29904	Eukaryotic Elongation Factor-2 Kinase	Protein kinase in the calmodulin-mediated signaling pathway that links activation of cell surface receptors to cell division	1.74	0.001
KCNG1	3755	Potassium Voltage-Gated Channel, Subfamily G, Member 1	Member of the potassium channel, voltage-gated, subfamily G	1.69	0.030
ETV7	51513	Ets Variant 7	Transcription factor; development and differentiation, oncogenesis, primarily in hematopoietic tissues	1.65	0.010
SMOX	54498	Spermine Oxidase	Cell cycle modulation, scavenging reactive oxygen species, and the control of gene expression	1.65	0.039
HIF3A	64334	Hypoxia Inducible Factor 3, Alpha Subunit	Transcription factor that regulates many adaptive responses to low oxygen tension (hypoxia)	1.64	0.010
ITGA9	3680	Integrin, Alpha 9	Forms an integrin that is a receptor for VCAM1, cytotactin and osteopontin	1.62	0.044
ARID3A	1820	AT Rich Interactive Domain 3A (BRIGHT-Like)	Important for normal embryogenesis, cell cycle control, transcriptional regulation	1.52	0.004
KLF15	28999	Kruppel-Like Factor 15	Transcriptional regulator; mediator of ER stress-induced insulin resistance, cardiac hypertrophy	1.50	0.027
GPSM1	26086	G-Protein Signaling Modulator 1	Influences the basal activity of G-protein signaling systems	1.50	0.041
ZMAT4	79698	Zinc Finger, Matrin-Type 4	Function not currenty well established	1.44	0.001

Gene	Betweenness Centrality	Degree (number of interacting partner)
CDH5	0.30	17
ESR1	0.28	19
CCR1	0.22	18
PCDH10	0.19	7
CCL5	0.13	14
GZMK	0.11	16
WISP2	0.09	3
IL2RB	0.08	15
SOX18	0.08	12
CFI	0.07	5
CALCRL	0.06	11
ERG	0.06	8
GNG11	0.06	8
KCNG1	0.06	5
TCF21	0.05	8
LCK	0.05	16
GPM6A	0.05	6
IGFBP5	0.05	5
PHF15	0.04	3
GIPC2	0.04	2
RPL12	0.04	9
VWF	0.04	9
EDN1	0.04	10
APEX1	0.03	4
ID1	0.03	6
CD79B	0.03	6
CCR10	0.02	5
SFRP4	0.02	5
PBX1	0.02	5
CCNA1	0.02	4
TSPAN7	0.02	3
STX1A	0.02	3
NRIP1	0.02	2
LGALS2	0.02	2
EEF2K	0.02	2
VWA5A	0.02	4
DUSP6	0.02	5
PGR	0.02	6
CD247	0.01	18
PRF1	0.01	6
DARC	0.01	3
KLRB1	0.01	8
ETV7	0.01	3
FBP1	0.01	5
CD3D	0.01	18
SLC2A5	0.01	2

CD3G	0.01	15
KLF15	0.01	3
TSPAN12	0.01	5
PTPRN2	0.01	3
NCAM2	0.01	3
THSD1	0.01	6
HERC6	0.01	3
FCGR3B	0.00	5
KLRD1	0.00	7
ZBTB16	0.00	2
ITK	0.00	13
CD8A	0.00	13
MAP4K1	0.00	7
HIF3A	0.00	3
ZAP70	0.00	10
TRAF3IP3	0.00	5
CLEC1A	0.00	5
ITGA9	0.00	3
ITPKA	0.00	3
GZMH	0.00	5
GNLY	0.00	5
CD6	0.00	4
PGBD5	0.00	4
RPS8	0.00	4
RAMP3	0.00	3
SH3KBP1	0.00	2
PRKD1	0.00	2
PMEPA1	0.00	2
PI15	0.00	2
KRT19	0.00	2
CCL23	0.00	2
CCL14	0.00	2
ZMAT4	0.00	1
ZFP69B	0.00	1
TNFRSF10A	0.00	1
TLL1	0.00	1
TIAM1	0.00	1
SYBU	0.00	1
SFRP1	0.00	1
RAMP2	0.00	1
RAMP1	0.00	1
PKIG	0.00	1
PDE10A	0.00	1
NR5A2	0.00	1
LIFR	0.00	1
ID3	0.00	1
ICA1	0.00	1

HOXB8	0.00	1
FOXP2	0.00	1
FOXP1	0.00	1
CYSLTR1	0.00	1
CORO2A	0.00	1
CCDC68	0.00	1
C1QL1	0.00	1
ATP5S	0.00	1
ATP5J	0.00	1
ASRGL1	0.00	1
ARHGEF3	0.00	1
ARHGEF18	0.00	1
ACACA	0.00	1
